# Adenosinergic Signaling as a Key Modulator of the Glioma Microenvironment and Reactive Astrocytes

**DOI:** 10.3389/fnins.2021.648476

**Published:** 2022-01-05

**Authors:** Gabriela N. Debom, Dominique S. Rubenich, Elizandra Braganhol

**Affiliations:** ^1^Programa de Pós-graduação em Biociências, Universidade Federal de Ciências da Saúde de Porto Alegre, Porto Alegre, Brazil; ^2^Instituto de Cardiologia do Rio Grande do Sul, Instituto de Cardiologia - Fundação Universitária de Cardiologia, Porto Alegre, Brazil

**Keywords:** tumor microenvironment, glioblastoma, tumor-associated astrocyte, A2-like astrocyte, adenosine, CD73

## Abstract

Astrocytes are numerous glial cells of the central nervous system (CNS) and play important roles in brain homeostasis. These cells can directly communicate with neurons by releasing gliotransmitters, such as adenosine triphosphate (ATP) and glutamate, into the multipartite synapse. Moreover, astrocytes respond to tissue injury in the CNS environment. Recently, astrocytic heterogeneity and plasticity have been discussed by several authors, with studies proposing a spectrum of astrocytic activation characterized by **A1**/neurotoxic and **A2**/neuroprotective polarization extremes. The fundamental roles of astrocytes in communicating with other cells and sustaining homeostasis are regulated by purinergic signaling. In the CNS environment, the gliotransmitter ATP acts cooperatively with other glial signaling molecules, such as cytokines, which may impact CNS functions by facilitating/inhibiting neurotransmitter release. Adenosine (ADO), the main product of extracellular ATP metabolism, is an important homeostatic modulator and acts as a neuromodulator in synaptic transmission via P1 receptor sensitization. Furthermore, purinergic signaling is a key factor in the tumor microenvironment (TME), as damaged cells release ATP, leading to ADO accumulation in the TME through the ectonucleotidase cascade. Indeed, the enzyme CD73, which converts AMP to ADO, is overexpressed in glioblastoma cells; this upregulation is associated with tumor aggressiveness. Because of the crucial activity of CD73 in these cells, extracellular ADO accumulation in the TME contributes to sustaining glioblastoma immune escape while promoting **A2**-like activation. The present review describes the importance of ADO in modulating astrocyte polarization and simultaneously promoting tumor growth. We also discuss whether targeting of CD73 to block ADO production can be used as an alternative cancer therapy.

## Introduction

In the last few years, astrocytes have received increased attention, with many studies aimed at in-depth understanding of their functions in the healthy brain and in central nervous system (CNS) pathologies (Khakh and Sofroniew, 2015; Escartin et al., 2019). Reactive astrocyte responses have been described as detrimental in different pathologies, including neuroinflammatory and neurodegenerative diseases and brain tumors (Liddelow and Barres, 2017).

In this regard, glioblastoma (GB) is the most common and aggressive primary tumor in the CNS (Huse and Holland, 2010). Although many researchers have attempted to develop new therapeutic strategies for GB, patients continue to show a short median survival time (Huse and Holland, 2010; Nørøxe et al., 2016; Di Carlo et al., 2019). The tumor microenvironment (TME) contains not only GB cells, but other normal cells such as immune cells and astrocytes that contribute to cancer progression and may shape the tumor cell response to radio-chemotherapy (Wang et al., 2017; Najafi et al., 2019; Wei et al., 2020). Therefore, GB-astrocyte crosstalk may explain the poor prognosis of patients by improving the understanding of how astrocytes contribute to GB progression, which can provide new biological treatment targets (Brandao et al., 2019).

The presence of extracellular adenosine triphosphate (ATP) and its hydrolysis products, namely ADP, AMP, and adenosine (ADO), triggers the purinergic signaling cascade (Burnstock, 2007; Di Virgilio et al., 2009). The biological effects of extracellular purines and pyrimidines are mediated by P1 and P2 purinoceptors (Zarrinmayeh and Territo, 2020). ADO mediates the sensitization of the P1 receptors named A_1_R, A_2A_R, A_2B_R, and A_3_R (Fredholm et al., 2011). The P2 family is subdivided into ionotropic P2X (1–7) and metabotropic P2Y (P2Y_1_, _2_, _4_, _6_, _11_, _12_, _13_, _14_) receptors (Burnstock, 2007; Di Virgilio et al., 2018). ATP plays a dual role in the TME by exhibiting anti- or pro-tumor effects depending on its extracellular concentration, the presence of purinergic receptors, and the combined activity of ectonucleotidases, such as ecto-nucleoside-triphosphate-diphosphohydrolases (E-NTPDases) and CD73, which metabolize ATP to ADO in the extracellular space (Allard et al., 2016, 2017). The amplitude of the agonist effects of ATP and its metabolite ADO is critical for maintaining the TME, as these signaling molecules have tumor-promoting activities in immune escape, angiogenesis, cell proliferation, and migration (Allard et al., 2016, 2020; Di Virgilio et al., 2016, 2018). In addition, extracellular ATP and ADO have important roles in neurodegenerative, cognitive, and psychiatric disorders. ADO modulates synapse function by altering neuron firing in different brain regions, contracting the local vasculature, and exerting immune/neuromodulatory effects (Dunwiddie, 1985; Redzic et al., 2010; Hinton et al., 2014; Allard et al., 2016; Di Virgilio et al., 2016). In this regard, selective participation of purinergic signaling in astrocytes has been well-established and recently reviewed (Illes et al., 2019; Agostinho et al., 2020; Lopes et al., 2021). However, astrocytic polarization has recently gained attention, although little is known about the contribution of extracellular purines to **A1**/neurotoxic or **A2**/neuroprotective astrocyte polarization. The present review summarizes the available evidence on the participation of purinergic signaling, mostly involving ADO, in modulating the astrocyte phenotype in the TME and its further impact on GB progression.

## Role of Reactive Astrocytes in the Brain

Astrocytes are abundant and complex glial cells in the CNS and are key elements involved in brain homeostasis (Sofroniew and Vinters, 2010). Astrocytes establish and maintain the blood–brain barrier with their cell end-feet (Ballabh et al., 2004; Verkhratsky and Nedergaard, 2018). Moreover, these glial cells are tightly integrated into neural networks, participating in synaptic transmission regulation via uptake of the neurotransmitter glutamate, and communicate with neighboring cells through Ca^2+^ signals (Clarke and Barres, 2013). Astrocytes form a fundamental part of synaptology together with the pre- and postsynaptic neuronal compartments as part of the multipartite synapse (Verkhratsky and Nedergaard, 2018).

Astrocytes undergo a set of morphological changes in their normal state in response to CNS insults (Wilhelmsson et al., 2006; Sofroniew, 2009; Burda et al., 2016; Liddelow et al., 2017). The reactivity of astrocytes is a universal reaction that occurs in response to cerebral injury. Under stress condition, these cells assume a wide range of new characteristics in brain, becoming hypertrophic, upregulating intermediate filaments such as nestin, vimentin, and glial fibrillary acidic protein, and in some cases, activating proliferation process (Pekny and Pekna, 2014; Boccazzi and Ceruti, 2016; Guan et al., 2018). Reactive astrocytes are observed in the brains of patients suffering from various pathological conditions, including trauma, infection, neurodegeneration, and ischemia (Zamanian et al., 2012).

**FIGURE 1:**
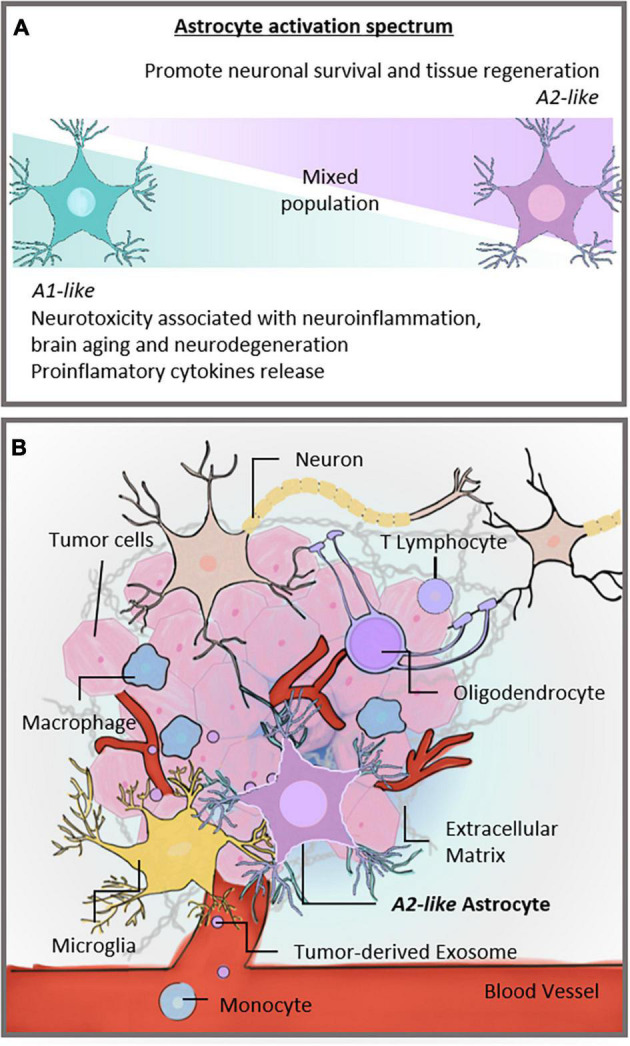
Activation spectrum of astrocytes and the brain TME. **(A)** Astrocytic spectrum of activation. Recent studies proposed that astrocytes became reactive and assumed an activation spectrum characterized by two extremes, the **A1** and **A2** phenotypes. The **A1**-astrocyte phenotype is associated with neurotoxic and neuroinflammatory effects, whereas the **A2**-astrocyte phenotype is related to neuroprotection. **(B)** Different cells constitute the TME of GBs. Neurons, microglia, and glial cells, such as astrocytes and oligodendrocytes, share the brain milieu with tumor cells, establishing a complex ecosystem. These cells are surrounded by extracellular matrix and susceptible to signals carried by tumor-derived exosomes. In addition, monocytes can be recruited to the brain site and contribute to forming the TME. Our hypothesis is that astrocytes in the TME assume an **A2**-like astrocyte phenotype, partly explaining GB malignancy. TME, tumor microenvironment; CNS, central nervous system; GB, glioblastoma.

Because of their variable roles in different pathological conditions, reactive astrocytes remain controversial. Although they are recognized as the pathological hallmark of CNS structural lesions, previous studies reported that, in addition to supporting CNS recovery, astrocytes could inhibit axon regeneration after CNS injury and produce proinflammatory cytokines that exacerbate neuroinflammatory damage (Sofroniew and Vinters, 2010; Zamanian et al., 2012; Liddelow et al., 2017). Recently, astrocytes were shown to react to insults in an activation spectrum, similar to that observed in macrophages and microglia in the CNS, assuming two opposite phenotypes in a spectrum of polarization: **A1**-astrocyte and **A2**-astrocyte (Liddelow and Barres, 2017; Figure 1A). **A1**-astrocytes are neurotoxic and associated with injuries and neurodegenerative pathologies such as Alzheimer’s disease (Goetzl et al., 2018; Carter et al., 2019; Grimaldi et al., 2019), Parkinson’s disease (Hinkle et al., 2019), and Huntington’s disease (Diaz-Castro et al., 2019). Moreover, **A1**-astrocyte polarization has been associated with normal brain aging, which supports the involvement of **A1**-astrocytes in neuroinflammation (Clarke et al., 2018). In contrast, **A2**-astrocytes tend to be neuroprotective and promote neuronal survival (Singh et al., 2019). Therefore, once induced, these two extremes of cell phenotypes diverge in gene expression, cell structure, signaling, and overall function (Escartin et al., 2019). The plasticity of astrocytes that enables them to exert diverse responses to injuries has become a topic of research interest, representing an opportunity to explore specific therapeutic strategies for brain pathologies including GB (Pavlou et al., 2019).

## Tumor Microenvironment of the Brain: The Importance of Microglia-Astrocyte Crosstalk

Glioblastoma is considered as the most common and aggressive primary brain tumor, accounting for more than 40% of neoplasms of the CNS (Huse and Holland, 2010; Di Carlo et al., 2019). Clinically, gliomas are divided into four degrees, grade I as benign, which show slow proliferation rate and good prognosis after surgical removal; grade II gliomas, which are slightly more severe and capable of progressing to higher grades; and grade III and IV gliomas, which are the most aggressive and characterized by a high rate of cell proliferation, spreading rapidly through the normal parenchyma of the CNS (Nørøxe et al., 2016; Verano-Braga et al., 2018). Moreover, GB (grade IV glioma) exhibits higher angiogenesis and necrosis when compared to grade I–III gliomas, resulting in a short survival time of approximately 15 months in patients (Huse and Holland, 2010; Di Carlo et al., 2019). The first-choice treatment for GB involves surgical removal combined with radio-chemotherapy with temozolomide (TMZ), a DNA-alkylating agent (Stupp et al., 2005).

The TME directly influences tumor growth and proliferation. The GB microenvironment comprises infiltrating and resident immune cells, vascular cells, and glial cells such as astrocytes and microglia (Chen and Hambardzumyan, 2018; Figure 1B). The interactions of tumor cells with non-malignant cells in the TME occur via direct cell-cell communication, shape tissue reorganization, and tumor ecosystem modulation (Tirosh and Suvà, 2018), and impact the biology and aggressiveness of GB (Azambuja et al., 2020a).

Tumor-associated macrophages are the dominant infiltrating immune cell population in the tumor mass, constituting ∼30–40% of total cells of the GB bulk (Hambardzumyan et al., 2016; Chen and Hambardzumyan, 2018). Microglia, as the resident innate immune cell of the CNS, is also abundant in the GB TME (Charles et al., 2011). Both macrophages and microglia display an alternative phenotype of activation or M2-*like* polarization when subverted by the tumor signals associated with growth, invasion, and angiogenesis, in addition to contributing to the establishment of an immunosuppressive environment (Da Fonseca et al., 2016; Placone et al., 2016; Matias et al., 2017; Roesch et al., 2018). In addition to macrophages and microglia, lymphocytes are recruited to the tumor site, a relevant part of the TME. A higher proportion of CD4^+^ than CD8^+^cells in the TME is associated with a high tumor grade and worst prognosis (Gieryng et al., 2017; Strepkos et al., 2020).

Other non-neoplastic cells are found in smaller numbers, such as oligodendrocyte precursor cells and neurons. Although there is limited evidence explaining the contribution of glial cells to immunosuppression, these cells have the potential to alter the TME (Henrik Heiland et al., 2019). Communication among these different cell types is an important process carried out by the exchange of information via extracellular vesicles, including exosomes, containing signaling proteins or regulatory RNAs (Rajendran et al., 2014).

The interaction of GB cells with astrocytes around the peritumoral area has become a topic of great interest. Reactive astrocytes exhibit intimate crosstalk with activated microglia and similar secreted factors such as interleukin-1β, interleukin-6, and nitric oxide, which are related to GB progression (Liddelow and Barres, 2017; Jha et al., 2019) and a poor prognosis for patients (Mega et al., 2020). This relationship may also be essential for the immune aspects of the brain tumor TME. Placone et al. (2016) suggested that astrocytes promote the proliferation and invasion of tumor cells and decrease chemotherapeutic efficiency by protecting malignant cells from apoptosis (Chen et al., 2015). Moreover, astrocytes cleave the inactive form of matrix metalloproteinase-2 (pro-MMP2) to matrix metalloproteinase 2, which is involved in tumor invasiveness (Le et al., 2003). Brandao et al. (2019) demonstrated that astrocytes are involved in GB progression, identifying reactive astrocytes around the tumor as tumor-associated astrocytes characterized by high proliferation, migration, and invasion to support tumor cell survival. Studies have demonstrated that astrocytes release exosome vesicles (EVs) containing promoters of angiogenesis and immune modulation, further supporting the novel capabilities of astrocytes in communicating with the TME (Proia et al., 2008; Hajrasouliha et al., 2013). Additionally, Leiss et al. (2020) highlighted astrocyte-glioma crosstalk, indicating that platelet-derived growth factor is a potential astrocytic biomarker associated with a poor prognosis in patients with GB.

Astrocytes in the peritumoral areas can be affected by GB, causing leakage through the blood–brain barrier and contributing to the entrance of a set of immune cells at the tumor site (Watkins et al., 2014; Nørøxe et al., 2016). The recruitment of new tumor-associated macrophages and regulatory T-cells to the TME and overall immunosuppressive microenvironment are crucial for GB development and progression and are factors that affect the poor response of this tumor to conventional immunotherapy (Joyce and Fearon, 2015; Broekman et al., 2018; Pasqualini et al., 2020).

In summary, tumors control their microenvironment to establish an immunosuppressed niche. The tumor capacity to “educate” non-transformed cells in the TME contributes to escape from surveillance by avoiding an effective immune response against tumor and preventing the recruitment of other adaptive and innate immune cells that may inhibit tumor growth (Da Fonseca et al., 2016). Therefore, tumor cells can effectively alter the immune system from a protective to a detrimental state and favor tumor progression (Chen and Hambardzumyan, 2018). In accordance with these observations, increasing evidence has revealed that astrocytes contribute to the immunosuppressive characteristics of the TME, and that microglia-astrocyte crosstalk is an important subject that requires further study (Placone et al., 2016). Besides that, the discussion about astrocytic activation states into the GB microenvironment needs to be amplified in order to comprehend the complexity of GB phenotypes and the diversity of the tumor ontogeny (Nørøxe et al., 2016).

## Purinergic Signaling in Astrocytes: From Adenosine Triphosphate to Adenosine

**FIGURE 2:**
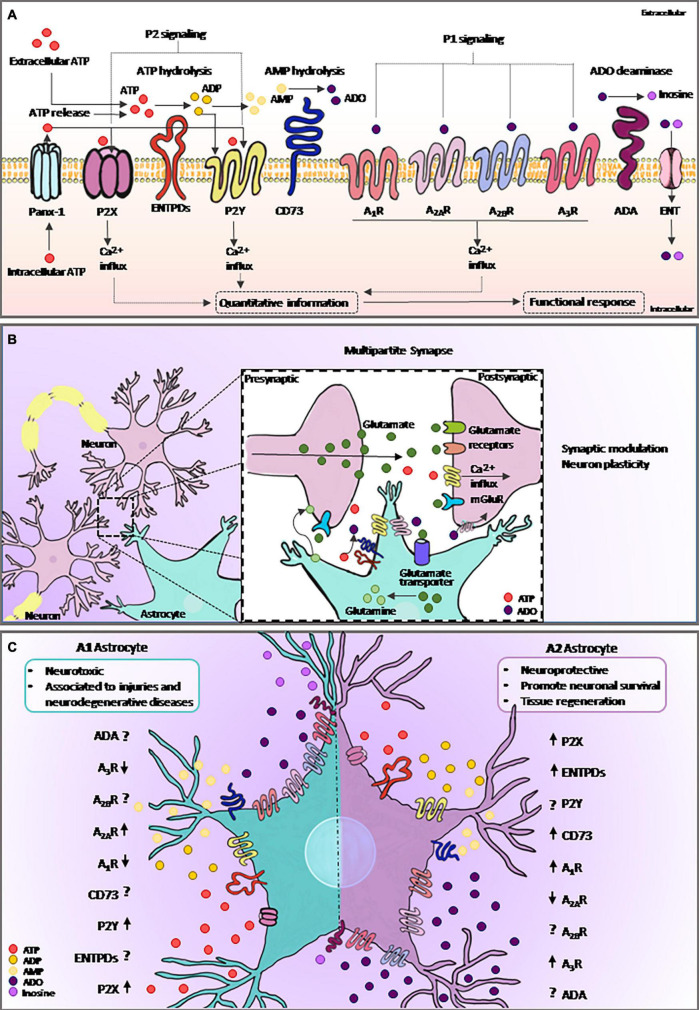
Purinergic signaling and astrocytes. **(A)** Intracellular ATP is released from astrocytes via specific channels such as Panx-1. Extracellular ATP and its breakdown products, ADP and ADO, are agonists of purinergic receptors, and the extracellular levels of nucleotides and nucleosides are regulated by ectonucleotidases such as ENTPDs and CD73. ADA catalyzes irreversible deamination of ADO into inosine. ADO can return to the intracellular space trough specific transporting channels, named as ENTs. Purinergic signaling plays an important role in many biological processes, including astrocytic functions. Both ATP released from astrocyte and from neighboring cells can selectively bind to P2 receptors, whereas ADO binds to P1 receptors. Activation of purinergic receptors in astrocytes can induce several functional responses, including the regulation of neural communication and immune/inflammatory responses. **(B)** ATP is an important gliotransmitter released by astrocytes in combination with glutamate. The multipartite synapse is a key mechanism of neuron communication and plasticity. **(C)** Astrocytes became reactive in response to injury. Here, we show the mechanism by which the elements of purinergic signaling are expressed or face this response. The graphical representation of purinergic signaling shows **A1**-astrocyte in the left panel and **A2**-astrocyte in the right panel. Arrows represent the described modulation of the elements found in reviewing the literature on the topic, and the interrogation points are represented as components without data already described in the literature. ATP, adenosine triphosphate; ADP, adenosine diphosphate; AMP, adenosine monophosphate; ADO, adenosine; Panx-1, pannexin channel 1; ADA, adenosine deaminase; ENTPDs, ecto-nucleoside-triphosphate-diphosphohydrolases; ENT, equilibrative nucleoside transporter.

ATP is known for its classic function in energy metabolism. However, in the extracellular environment, this nucleotide is crucial for the maintenance of physiological functions including neuro/gliotransmission and immune/inflammatory responses (Khakh and Burnstock, 2009). ATP can be metabolized to ADO by ectonucleotidases, such as CD39 (NTPDase1) and CD73 (ecto-5′-nucleotidase), which play key roles by producing active hydrolyzed products and recycling nucleotides (Burnstock, 2007; Allard et al., 2017). Adenosine deaminase (ADA) is responsible for converting ADO to inosine, and both ADO and inosine can be transported across cellular membranes through nucleoside transporters (ENTs) (Boswell-Casteel and Hays, 2017; Pastor-Anglada and Pérez-Torras, 2018; Figure 2A).

ATP is considered as a danger-associated molecule that is released by injured cells into the extracellular medium during inflammation (Minkiewicz et al., 2013). In addition, ATP can be released by astrocytes through specific and regulated pathways such as exocytosis (Pangršič et al., 2007), vesicular release (Coco et al., 2003; Zorec et al., 2016), or diffusion through ion channels, specifically pannexin/connexin channels (Stout et al., 2002; Suadicani et al., 2012). Increasing evidence has demonstrated that both astrocytes and tumor cells express purinergic receptors and purine-metabolizing enzymes, which can trigger a set of biological effects in the CNS (Wink et al., 2003, 2006; Rodrigues et al., 2015; Azambuja et al., 2019a; Illes et al., 2019; Campos-Contreras et al., 2020). In line with this, ATP and ADO signaling may have significant impacts on therapies for brain tumors such as GB (Azambuja et al., 2019a,b, 2020c).

Astrocytes release various gliotransmitters, such as glutamate, D-serine, and ATP (Figure 2B). Notably, after activation, a single astrocyte releases both glutamate and ATP in the multipartite synapse, leading to biphasic modulation of synaptic transmission (Ma et al., 2016). Extracellular ATP is well-known for its role in mediating astrocytic Ca^2+^ waves (Guthrie et al., 1999) as well as in astrocyte-mediated wound healing in glial scars (Singh et al., 2015). Moreover, the catabolism of ATP likely represents the source of ADO inactivating postsynaptic A_2A_R (Agostinho et al., 2020), as well as the mechanism by which the release of gliotransmitters by astrocyte exerts neuronal feedback actions and eventually modulates synaptic transmission and plasticity (Illes et al., 2019; Scemes et al., 2019).

As described above, microglia-astrocyte crosstalk is instrumental to CNS functions and determines the fate of astrocytes and microglia activation (Jha et al., 2019). Microglial activation by lipopolysaccharide, a TLR4 agonist, induces the release of ATP, which in turn stimulates P2Y_1_ in astrocytes, leading to the release of glutamate and further modulation of neuronal activity (Pascual et al., 2012). Cortical astrocytes respond to extracellular ATP under *in vitro* experimental conditions, and astrocytic activation into a reactive phenotype, including cell proliferation and glial fibrillary acidic protein remodeling, depends on ATP levels (Suadicani et al., 2012). Similarly, Ficker et al. (2014) found a correlation between P2X7 receptor sensitization and the development of inflammatory and neuropathic pain, possibly involving oxidative stress.

ATP can be converted into its breakdown product ADO in the extracellular environment by ectonucleotidases. A previous study reported a purinergic enzyme profile for E-NTPDases and CD73 in rat astrocytes, demonstrating that extracellular ATP is rapidly converted to ADP, and ultimately, to AMP by NTPDase2, an ectoenzyme predominantly expressed in these cells (Wink et al., 2003, 2006). As high levels of extracellular ATP are neurotoxic, NTPDase2 activity in astrocytes may protect neurons from damage, in addition to promoting ADO production (Wink et al., 2006). In addition, CD73 overexpression was observed in reactive astrocytes as a possible protective astrocyte repertoire during the symptomatic phase of an experimental autoimmune encephalomyelitis model, demonstrating the relationship between the ATP/ADO ratio and acute inflammatory situations (Lavrnja et al., 2015). CD73 activity is also involved in astrocyte adhesion and migration, possibly via interactions with the extracellular matrix (Adzic et al., 2017; Adzic and Nedeljkovic, 2018).

In general, ADO is directly related to cellular processes such as viability and adaptability (Cunha, 2016). However, under pathological conditions, the ADO concentration acts as a risk factor for CNS pathologies, including the development/progression of neurodegenerative diseases and brain tumors (Cunha, 2016; Azambuja et al., 2020e). Thus, studies have focused on investigating astrocyte plasticity to understand the role of ADO in pathological situations.

The ability of astrocytes to detect patterns of neural activity is attributed to the fine adjustment of astrocyte signaling, which in turn modulates synaptic transmission and contributes to the most diverse forms of synaptic plasticity (Covelo and Araque, 2018; Mederos et al., 2019). Brain injuries caused by trauma or stroke increase extracellular ADO levels (Haskó et al., 2005; Fusco et al., 2018). In this regard, the P1 receptors A_1_R and A_2A_R are located at synapses, particularly at excitatory ones, and cross-communication between astrocytes and neurons regulates the neural networks (Rebola et al., 2008; Illes et al., 2019). Indeed, astrocytes depress excitatory synapses and potentiate inhibitory synapses by activating A_1_R and A_2A_R, respectively (Martin-Fernandez et al., 2017). Interestingly, astrocytic A_2A_R modulates glutamate-mediated signaling through a variety of mechanisms (Rebola et al., 2008). For example, A_2A_R influences glutamate and GABA uptake by neurons, and selective A_2A_R activation in astrocytes inhibits glutamate uptake by decreasing Na^+^/K^+^-ATPases (NKAs) and the α2 subunit of NKA (Matos et al., 2013). Based on these results, A_2A_R overexpression observed in astrocyte reactivity contributes to the progression of brain diseases, making this receptor a potential therapeutical target for Alzheimer’s disease, Parkinson’s disease, and Sandhoff disease, as previously reported and extensively revised (Ohta et al., 2006; Matos et al., 2015; Scharbarg et al., 2016; Basanta and Anderson, 2017; Liddelow and Barres, 2017; Quail and Joyce, 2017; Wesseling and Capper, 2018; Allen et al., 2019; Okada et al., 2019; Schalla and Stengel, 2019; Azambuja et al., 2020d; data summarized in Table 1).

**TABLE 1:** Purinergic signaling in astrocytes.

Nucleotide or nucleoside/receptor/ channel/enzyme	Biological process	Experimental system	Effect	References
ATP	Neuroinflammation	*In vitro*	Induction of astrocytic reactivity by ATP	Adzic et al., 2017
ATP	Neuron and glia plasticity	*In vitro*	ATP released as an activation process of astrocytes	Singh et al., 2015
ATP/ADO	Neuron and glia plasticity	*In vivo*	ATP/ADO are released downstream of the GABA-mediated astrocyte Ca^2+^ signal	Covelo and Araque, 2018
Panx1	Neuron and glia plasticity	*In vivo*	Related to ATP release in astrocytes and better outcome of seizures	Scemes et al., 2019
P2X7	Neuroinflammation and pain	*In vivo*	P2X7 receptors as a key mechanism in inducing pain during inflammation	Ficker et al., 2014
CD73	Cell adhesion/migration	*In vitro*	Upregulation of CD73	Adzic and Nedeljkovic, 2018
CD73	Neuroinflammation	*In vivo*	Neuroprotection of CD73 overexpression during acute inflammation	Lavrnja et al., 2015
ADO	Neuron and glia plasticity	Cerebral cortex human astrocytes	Inhibition of astrocyte proliferation independent of P1 receptor sensitization	Marcelino et al., 2020
P2Y_1_ + A_2A_R	Neuron and glia plasticity	*In vivo*	ATP/ADO release impact synaptic plasticity and enhance cognitive functions	Mederos et al., 2019
rENT-1	Neurodegeneration	*In vitro*	Neuroprotection role of ADO after hypoxia and glucose deprivation	Redzic et al., 2010
ENT1	Neurodegeneration	*In vitro* + *In vivo*	Reduced GFAP expression in astrocyte cultures ENT1-knockdown, and in ENT1^–/–^mice	Hinton et al., 2014
AR	Neuron and glia plasticity	*In vivo (Drosophila)*	Neuromodulation by ATP release from astrocytes and subsequent activation of AR on dopaminergic neurons	Ma et al., 2016
A_1_R	Neuron and glia plasticity	*In vitro* + *In vivo*	Activation of A_1_R receptors is increased by wakefulness	Schmitt et al., 2012
A_1_R	Neuron and glia plasticity	*In vivo*	Astrocytic ATP and ATP-derived adenosine involved in the cognitive deficits following sleep deprivation	Florian et al., 2011
A_1_R	Neuroinflammation	*In vivo*	Induction of inflammation via LPS and control of sleep	Nadjar et al., 2013
A_1_R	Neuroinflammation	*In vivo*	Neuroprotective effects	Kong et al., 2020
A_1_R – A_2A_R	Neuron and glia plasticity	*In vivo*	Neuromodulation by regulating specific synapses via ATP/ADO	Martin-Fernandez et al., 2017
A_2B_R	Neuron and glia plasticity	*In vitro*	Role of ADO in neuronal protection	Moidunny et al., 2012
A_2A_R	Neuron and glia plasticity	*In vitro*	Increase in extracellular glucose concentration induces astrocytic ADO release, regulating the need for sleep	Scharbarg et al., 2016
A_1_R-A_2A_R	Neuroinflammation	Surgical specimens from glioma patients	Neuroprotective and anti-convulsive effect	Huang et al., 2016
A_2A_R	Neurodegeneration	*In vivo*	Neuroprotection after A_2A_R antagonism using MSX-3	Cerri et al., 2014
A_2A_R	Neuron and glia plasticity	*In vivo*	Interaction between A_2A_R and D2-dopamine receptors	Cervetto et al., 2018
A_2A_R	Neuron and glia plasticity	*In vivo*	Dysfunction of astrocytic A_2A_R triggers the crosstalk between astrocyte and neuron	Matos et al., 2015
A_2A_R	Neuroinflammation	*In vitro* + *In vivo*	Astrocytic activation via A_2A_R as an important mediator of inflammation mediated by microglial activation	Ogawa et al., 2018
A_2A_R	Neuron and glia plasticity	*In vitro*	Astroglial glutamatergic transmission	Okada et al., 2019
A_2A_R	Neurodegeneration	*In vitro*	Overexpression upregulates astrocytic genes related to aging and astrocytic reactivity	Paiva et al., 2019
A_2A_R – A_2B_R	Neuron and glia plasticity	*In vitro*	Activation of receptors in affecting synaptic networks and neuronal activity	Eusemann et al., 2015
A_2B_R	Neurodegeneration	*In vivo*	ADO as a neuroprotector	Fusco et al., 2018
ADA	Neurodegeneration	*In vitro*	Decrease of ADA activity	Allen et al., 2019

*ADA, adenosine deaminase; ADO, adenosine; AR, adenosine receptor; ATP, adenosine triphosphate; ENT1, equilibrative nucleoside transporter 1; LPS, lipopolysaccharide; Panx1, Pannexin 1; rENT1, rat equilibrative nucleoside transporter 1.*

Although A_2A_R has shown a protagonist effect in studies of neurodegenerative disease, some neuroprotective actions have been reported for A_2B_R sensitization. Moidunny et al. (2012) observed a positive correlation among astrocytic leukemia inhibitory factor protein expression, A_2B_R sensitization, and further Gq/11-PLC-PKC-MAPK-NFκB cascade activation, supporting the neuroprotective effect of A_2B_R under excitotoxic conditions. In contrast, in an ischemia model, the use of A_2B_R antagonists (MRS1754 and PSB603) partially limited astrocyte proliferation, thus preventing neurodegenerative effects on neurons (Fusco et al., 2018).

In addition to the relation with astrocytes, purinergic signaling is also an important player in the microglial reactivity. In the CNS, the P2X7 receptor is preferentially located on microglia (Illes, 2020). Besides that, several authors have also been reported the expression of P1 receptors in microglia, describing ADO as a crucial modulator of microglia phenotype of CNS pathologies, including cancer (Hammarberg et al., 2003; Synowitz et al., 2006; Ferreira-Silva et al., 2020).

Extracellular ADO is ultimately converted to inosine by ADA, which exerts many biological regulatory functions such as protecting the brain against neuronal diseases. A very informative study applied an experimental strategy for profiling fibroblasts and inducing neuronal progenitor-derived human-induced astrocytes from patients with amyotrophic lateral sclerosis, to evaluate dysfunctional astrocytic energy metabolism. The investigation revealed that induced human astrocytes had reduced ADA activity, making the astrocytes more susceptible to ADO-induced toxicity. In contrast, restoration of ADA activity and/or supplementation with inosine stimulated the aerobic state of astrocytes, increased the bioenergetic capacity, and decreased neurotoxicity, thereby demonstrating a beneficial therapeutic approach (Allen et al., 2019).

Considering the collective results of previous studies, A_1_R, A_2B_R, and ADA activity in astrocytes is crucial for neuronal maintenance and may contribute to the **A2**/neuroprotective profile of astrocytes. In contrast, overexpression of A_2A_R disrupts adenosinergic homeostasis and is involved in neuronal degeneration, representing a classic condition of **A1/**neurotoxic activation in astrocytes. These data are summarized in Table 1. An imbalance of the adenosinergic pathway is common in neuronal disorders. ADO exerts a strong inhibitory or excitatory influence on neuronal synapses. Notably, neuronal-stem cell-derived astrocytes can develop into GB (Yao et al., 2018). Therefore, it is essential to understand the activation mechanisms of astrocytes and influence of ADO on their proliferation and function.

## Adenosinergic Signaling in Glioblastoma Microenvironment

Considering the lack of effective therapies for treating patients with GBs, new biological targets must be identified, and adenosinergic signaling has emerged as a candidate target (Azambuja et al., 2019a,b, 2020c,d, 2020e; Yan et al., 2019). Studies by our group reported that CD73 overexpression in GB favors tumor progression (Azambuja et al., 2019b,c, 2020d). Moreover, A_1_R sensitization potentiates *in vitro* GB cell proliferation, migration, and invasion and contributes to TMZ resistance (Azambuja et al., 2019a). The importance of this protein in cell adhesion and invasion has been verified, as it was observed that CD73 in GB interacts with the extracellular matrix of the TME (Cappellari et al., 2012b). Using an astrocytoma cell line (U373), A_2B_R was described as a low-affinity receptor activated only by high concentrations of ADO, which occurs in pathological conditions such as hypoxia, and in contrast, A_2A_R was expressed under physiological ADO levels in the brain (Eusemann et al., 2015). Studies of CD73-FLK mice detected positive regulation of A_2B_R in GB, and its blockage potentiated TMZ-induced tumor cell death (Yan et al., 2019). A_1_R and A_2A_R are highly expressed in high-grade gliomas, specifically in grade III astrocytoma. In low-grade gliomas, A_1_R and A_2A_R exhibit low expression. ADO acts as a neuroprotective agent and prevents hypoxic toxicity, that is, deregulation of A_1_R and A_2A_R axis influences the metabolism of ADO in the invasive process of GB (Huang et al., 2016).

The tumor niche contains sites of hypoxia formed by the high rate of cell proliferation, without a corresponding increase in the rate of new blood vessel formation. This condition contributes to increased extracellular ATP levels, and consequently, selects the population of immunosuppressive cells present in the TME (Trabanelli et al., 2012). Interestingly, hypoxia conditions and the subsequent release of hypoxia-inducible factor-1α have been associated with the accumulation of extracellular ADO in the TME via the CD39-CD73 axis and further signaling through A_2A_R (Hatfield et al., 2014). Torres et al. (2019) reported that extracellular ADO production was higher in hypoxia conditions to promote cell migration and invasion in a hypoxia-inducible factor-2 dependent process, whereas A_3_R blockade reversed this effect. This corroborated the findings of Niechi et al. (2019), who showed that ADO depletion decreases tumor aggressiveness. In addition, clinical trials of cancer immunotherapy targeting the “hypoxia-adenosinergic pathway” through hyperoxic breathing stimulation and A_2A_R blockage in combination with conventional anti-PD1/PDL1 immunotherapy are underway (Ohta et al., 2006; Hatfield et al., 2015; Hatfield and Sitkovsky, 2020). These trials are reporting promising results in patients refractory to current therapies (Fong et al., 2020; Willingham et al., 2020). However, it is important to highlight the lack of studies on the therapeutic effect in human CNS tumors.

The release of EVs from tumor cells supports the ability of GBs to communicate with distant cells. Interestingly, the content of EVs reflects the activation state of the mother cell; hence, tumor cells can proliferate and modulate acceptor cells (Matarredona and Pastor, 2019; Zhang et al., 2019; Schuurmans et al., 2020). Studies of EVs are emerging and have shown promising results regarding their ability to maintain a propitious niche for tumor development, as they can modulate immune, epithelial, and glial cell signaling (Benito-Martin et al., 2015; Whiteside, 2017; Almeida et al., 2019; Boomgarden et al., 2020). Particularly, glioma cell lines as well as primary cultured cells released EVs capable of promoting M2-like activation of tumor-associated macrophages (Azambuja et al., 2020b). Moreover, exosomes, a subclassification of EVs, carry ADO and its products, such as inosine, hypoxanthine, and xanthine, which further contribute to independent adenosinergic signaling (Azambuja et al., 2020a; Ludwig et al., 2020).

Among the non-neoplastic cells comprising the TME, astrocytes stand out because of their high phenotypic similarity to GB cells (Galland et al., 2019). As mentioned earlier in this review, the astrocyte spectrum of activation of the **A2**-like phenotype is associated with the success of GB progression. Astrocyte phenotypic modulation can be attributed to GB, further supporting the invasion and resistance to therapy (Oushy et al., 2018; Hallal et al., 2019). One of the main functions of ADO in the extracellular environment is to act as a potent immunosuppressive mediator, which benefits tumor progression (Linden, 2011; Tozaki-Saitoh et al., 2011; Allard et al., 2016; Arab and Hadjati, 2019).

Therefore, in agreement with the results of studies using other non-transformed cells comprising the TME, such as macrophages and microglia, the purinergic receptors and ectonucleotidases may be integral components affecting the phenotypic differentiation of tumor-associated astrocytes (Figure 2C). A better understanding of the crosstalk among microglia, astrocytes, and tumor cells may reveal innovative therapeutic options based mainly on adenosinergic signaling to overcome GB progression.

## Adenosinergic Signaling as a Therapeutical Strategy for Glioblastoma

Considering the present review, it is possible to understand the importance of astrocytes in maintaining homeostasis in the brain and the effective participation of ATP and ADO signaling in several processes related to health and disease. As the main source of ADO in the CNS is extracellular ATP metabolism, regulation of the hydrolysis of AMP to ADO may play a crucial role in maintaining GB-associated immunosuppression and may be an additional alternative for GB treatment in combination with standard radio- and chemotherapy with TMZ. Indeed, studies have shown the potential of P1 receptor antagonism for anti-glioma therapy. For example, the A_2B_R blockage would contribute to recovering GB chemosensitivity to TMZ (Yan et al., 2019). In addition, the pharmacological blockade of CD73 using a selective inhibitor, the α,β-methylene adenosine 5′-diphosphate (APCP) have been described as a potential therapy for several tumors, such as melanoma, breast cancer, and gliomas (Koszałka et al., 2014; Tomczyk et al., 2018).

**FIGURE 3:**
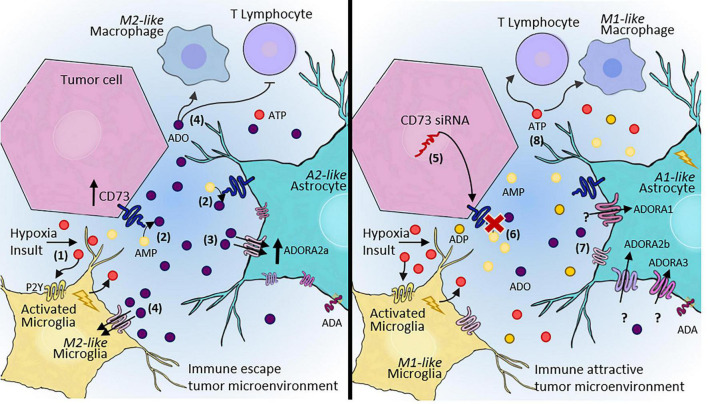
Adenosinergic signaling as a therapeutic target for GB treatment. **(1)** After hypoxia or other insults, microglia and astrocytes become reactive, contributing to the release of ATP into the extracellular space. **(2)** Our hypothesis is that rapid conversion of ATP to ADO via CD73 activity expressed by both tumor cells and **A2**-astrocytes in the TME promote immune escape. **(3)** ADO can sensitize P1 receptors, mainly A_2A_R, contributing to the maintenance of escape from immune surveillance, and consequently, increasing tumor malignancy and progression. **(4)** ADO promotes an immunosuppressive TME, which inhibits T lymphocyte recruitment and induces M2-like macrophage/microglia polarization. **(5)** In contrast, a therapeutic approach blocking CD73 may alter this entire pathway, **(6)** leading to decreased extracellular ADO. **(7)** A decreased ADO concentration in the TME may contribute to A1-astrocyte polarization and consequent impairment of tumor growth. **A1**-astrocytes overexpress A_1_R, A_2B_R, and A_3_R, but this effect has not been elucidated in tumors. **(8)** In summary, a therapeutic approach aimed at blocking adenosinergic signaling would establish an immune-attractive tumor microenvironment, resulting in the recruitment of effective immune cells to combat tumor cells. ATP, adenosine triphosphate; AMP, adenosine monophosphate; ADO, adenosine; siRNA, small interfering RNA; TME, tumor microenvironment.

By using CD73 as a molecular target and nanotechnology as a strategy for siRNA delivery to the CNS, our group demonstrated the potential of CD73 inhibition and knockdown for controlling *in vitro* and *in vivo* tumor progression (Azambuja et al., 2019a). According to our studies, the pharmacological inhibition as well as the silencing of CD73 impaired the protumor activities of CD73, decreasing the tumor volume and providing an opportunity to overcome chemoresistance and/or improve the TMZ effect (Azambuja et al., 2019a,2020c,2020d). We also recently reported the impact of blocking CD73 and consequent decrease in ADO availability on the levels of M2-like macrophages in the TME in a preclinical rat GB model (Azambuja et al., 2020e; Figure 3). These data strongly indicate the potential of using therapeutics to modulate the TME via purinergic constituents to re-establish the antitumor activity of immune cells. Nonetheless, the CD73 inhibition may not be always therapeutically helpful for tumors as seen for the GB and the specific biological characteristics of each tumor should be considered. For example, in medulloblastoma, the CD73 expression is a marker associated with better prognosis of patients (Cappellari et al., 2012a,^2015^).

Considering the similar features and responses of microglia and astrocytes in the TME, the ADO pathways may also be altered in astrocytic polarization, providing a therapeutic approach for treating patients with GB. Moreover, the participation of ADO and P1 receptors in the blood-brain barrier-associated properties is another interesting point for discussion about the therapeutical potential of ADO signaling blockage. As extensively described by Bynoe et al. (2015), both astrocyte’s end-feet and endothelial cells of the blood-brain barrier express those receptors, especially A_1_R and A_2A_R. Therefore, P1R mediated-signaling is decisive to regulate the permeability of macromolecules, inflammatory cells, and even therapeutic drugs/cells into the CNS (Bynoe et al., 2015).

## Concluding Remarks

The present review raises the hypothesis that reactive astrocytes are involved in the progression of brain tumors, such as GB. As astrocytes are instrumental to the microenvironment of the CNS, they can be corrupted by tumor cells and directly and indirectly participate in the TME, thus regulating crosstalk among tumor, glial, and endothelial cells.

Notably, CD73 knockdown or inhibition decreases *in vitro* and *in vivo* GB growth, and CD73 is an interesting target for brain tumor therapy. Additionally, adenosinergic therapy can be applied in most TME cells to inactivate not only transformed cells but also tumor-associated cells, including A2-astrocytes that support tumor progression.

## Open Questions

Considering the recent advances described in this review, although astrocyte participation in healthy and diseased brain biology has been well-established, there is no general agreement on astrocytic polarization in different phenotypes according to the nature of the injury, nor specifically in brain tumors. Recently, astrocytes were proposed to be involved in neuroinflammation. Further studies are needed to better describe their full characteristics and key features in different disorders involving immune components. Moreover, studies of astrocytic polarization are necessary to understand if different injury conditions lead to the production of different subpopulations of **A1**/neurotoxic and **A2**/neuroprotective astrocytes, or to transition stages until the fully reactivated cell state is reached.

Few studies have focused on the link between purinergic signaling and astrocyte participation in the tumor niche constitution. Considering the key role of the gliotransmitter ATP and its metabolite ADO on astrocyte functions, the specific factors involved in purinergic signaling, particularly adenosinergic signaling, as potential makers of astrocyte polarization should be further examined. Additionally, the contribution of ADO as a potent modulator of immune responses may directly influence astrocytic polarization, which requires further analysis. Moreover, the contribution of astrocyte-microglia crosstalk may be another key element in the outcome of GB, as well as for other CNS pathologies.

Finally, considering the genetic and phenotypic similarities of astrocytes and GB cells, tracing possible molecular markers or determining the signature of different astrocytic phenotypes is a crucial strategy for determining the contribution of non-malignant astrocytes in brain tumor physiology and establishing more effective therapeutic approaches.

## Author Contributions

GD and DR selected articles in PubMed and analyzed articles. GD created Table 1. DR assembled the figures. EB acquired the funding, supervised, and guided the entire work. All authors conceived the idea of studying the role of purinergic signaling in astrocytes and tumor progression, wrote and edited the article, and reviewed and edited the final text.

## Conflict of Interest

The authors declare that the research was conducted in the absence of any commercial or financial relationships that could be construed as a potential conflict of interest.

## Publisher’s Note

All claims expressed in this article are solely those of the authors and do not necessarily represent those of their affiliated organizations, or those of the publisher, the editors and the reviewers. Any product that may be evaluated in this article, or claim that may be made by its manufacturer, is not guaranteed or endorsed by the publisher.
